# Biomarkers in cardiogenic shock: old pals, new friends

**DOI:** 10.1186/s13613-024-01388-x

**Published:** 2024-10-16

**Authors:** Mathieu Jozwiak, Sung Yoon Lim, Xiang Si, Xavier Monnet

**Affiliations:** 1 Service de Médecine Intensive Réanimation, CHU de Nice, Hôpital L’Archet 1, 151 Route Saint Antoine de Ginestière, 06200 Nice, France; 2 UR2CA, Unité de Recherche Clinique Côte d’Azur, Université Côte d’Azur, 06200 Nice, France; 3 AP-HP, Service de Médecine Intensive-Réanimation, Hôpital de Bicêtre, DMU 4 CORREVE, Inserm UMR S_999, FHU SEPSIS, CARMAS, Université Paris-Saclay, 78 Rue du Général Leclerc, 94270 Le Kremlin-Bicêtre, France; 4 Division of Pulmonary and Critical Care Medicine, Department of Internal Medicine, Seoul National University Bundang Hospital, Seongnam, Korea; 5 Department of Critical Care Medicine, First Affiliated Hospital, Sun Yat-Sen University, Guangzhou, China

**Keywords:** Lactate, Troponin, B-type natriuretic peptide, Adrenomedullin, Dipeptidyl peptidase-3, Soluble suppression of tumorigenicity 2 receptor

## Abstract

In cardiogenic shock, biomarkers should ideally help make the diagnosis, choose the right therapeutic options and monitor the patient in addition to clinical and echocardiographic indices. Among “old” biomarkers that have been used for decades, lactate detects, quantifies, and follows anaerobic metabolism, despite its lack of specificity. Renal and liver biomarkers are indispensable for detecting the effect of shock on organ function and are highly predictive of poor outcomes. Direct biomarkers of cardiac damage such as cardiac troponins, B-type natriuretic and N-terminal pro-B-type natriuretic peptides have a good prognostic value, but they lack specificity to detect a cardiogenic cause of shock, as many factors influence their plasma concentrations in critically ill patients. Among the biomarkers that have been more recently described, dipeptidyl peptidase-3 is one of the most interesting. In addition to its prognostic value, it could represent a therapeutic target in cardiogenic shock in the future as a specific antibody inhibits its activity. Adrenomedullin is a small peptide hormone secreted by various tissues, including vascular smooth muscle cells and endothelium, particularly under pathological conditions. It has a vasodilator effect and has prognostic value during cardiogenic shock. An antibody inhibits its activity and so adrenomedullin could represent a therapeutic target in cardiogenic shock. An increasing number of inflammatory biomarkers are also of proven prognostic value in cardiogenic shock, reflecting the inflammatory reaction associated with the syndrome. Some of them are combined to form prognostic proteomic scores. Alongside clinical variables, biomarkers can be used to establish biological “signatures” characteristic of the pathophysiological pathways involved in cardiogenic shock. This helps describe patient subphenotypes, which could in the future be used in clinical trials to define patient populations responding specifically to a treatment.

## Introduction

Cardiogenic shock (CS) is a complex syndrome, which involves multiple pathophysiological pathways and affects numerous organs and systems. In recent years, the incidence of CS related to acute myocardial infarction has decreased, in contrast to non-ischemic causes [1–3]. Nowadays, patients with CS represent less than 10% of patients admitted to critical care [4], including medical and medical-surgical units or coronary care units, and this proportion varies according to the type of critical care unit. Their mortality rate has been unchanged over the last 10 years [1, 2] and is similar to or even higher than that of patients with septic shock [4]. The management of CS still represents a complex challenge, requiring the implementation of several simultaneous treatments, including mechanical support in most severe cases.

In this review, we describe the most recent developments regarding biomarkers in the field of CS, reviewing their pathophysiological significance, and diagnostic and prognostic value. Any biochemical variable can be considered a biomarker. We will focus on those of established or possible clinical interest. We will discuss the classic variables long used to characterize cellular metabolism or to describe cardiac damage. We will also consider biomarkers that are less specific to the heart, but which reflect the neurohormonal and inflammatory disorders accompanying CS. We will see how recent studies have used biomarkers to determine patient subphenotypes, homogeneous in their characteristics and prognosis. Finally, we will discuss the value of biomarkers for clinical practice.

## Lactate

During aerobic metabolism, glucose is converted to pyruvate, which is decarboxylated to acetyl-CoA, which enters the Krebs cycle. When aerobic metabolism slows down, the intracytoplasmic concentration of pyruvate increases. Consequently, lactate formation by lactate dehydrogenase increases. The elevation of plasma lactate concentration is therefore a very sensitive marker of anaerobic metabolism [5–7]. However, lactate poses two major problems in clinical practice. First, plasma concentration increases in many situations despite the absence of anaerobic metabolism: overproduction of pyruvate (beta-agonist, respiratory alkalosis, hemopathy), decrease in pyruvate utilization (mitochondrial dysfunction, drug-induced or congenital disease), or reduction in lactate clearance (liver or renal failure) [5]. Second, changes in plasma lactate concentrations are delayed [8], which prevents immediate assessment of the effects of therapeutic interventions. Despite these two limitations, lactate remains widely used in clinical practice in all patients with circulatory failure, including CS, to detect anaerobiosis, quantify it and monitor the effects of therapies. In CS, increased lactate concentrations are due to anaerobic metabolism, but may also be provoked by acute kidney injury, liver failure and catecholamine infusion. Given the lack of specificity of lactate as a marker of anaerobic metabolism, it must be interpreted considering markers of tissue perfusion, including capillary refill time, macrohemodynamic variables like cardiac output and arterial pressure, and venous oxygen saturation which indicates the balance between oxygen delivery and consumption, and which we will not detail here.

An increased plasma lactate concentration is clearly linked to the prognosis of patients with CS [9, 10] as also of other critically ill patients [7]. As during septic shock, changes in lactate, often incorrectly referred to as “lactate clearance”, could have a prognostic value greater than just the value at admission [11]. In a sub-analysis of the Danish IAPB-SHOCK II cohort including patients with CS related to acute myocardial infarction, the lactate value at 8 h was better at predicting 30 day mortality than its baseline value, with a cut-off value of 3.1 mmol/L [12]. In patients with septic shock, the ANDROMEDA shock study showed that targeting the normalization of the capillary refill time was not more effective in terms of mortality than targeting a lactate clearance ≥ 20% per 2 h [13]. No study has so far compared peripheral perfusion-targeted resuscitation with lactate-targeted resuscitation strategy in patients with CS.

## Renal and liver biomarkers

Acute kidney injury is an obvious marker of the severity of CS. The elevation of plasma creatinine concentration is included in the two risk scores currently used in CS, the CardShock [14] and the IABP-Shock score [15]. It seems that the new markers of renal function do not provide any prognostic advantage. In a subgroup of patients of the IABP-SHOCK II study, plasma concentrations of neutrophil gelatinase-associated lipocalin, kidney injury molecule-1, or cystatin C, and calculated glomerular filtration rate were not superior to simple plasma creatinine concentration in predicting 1 year mortality [16].

Liver damage, resulting from congestion or hypoperfusion, is also part of the management of patients with acute heart failure. The plasma concentrations of alanine amino-transferase (ALT) and aspartate amino-transferase (AST) on admission are associated with mortality during CS [17] as well as during myocardial infarction [18]. Changes in AST and ALT are also important to consider. A study of 178 patients with CS showed that in multivariate regression analysis, an increase in ALT > 20% during the first 24 h was associated with an increase in 90 day mortality, independently of other risk factors [17].

## Direct biomarkers of cardiac damage

### Cardiac troponins

These protein complexes modulate the sensitivity of muscle cells to calcium. They are made up of three different proteins (troponin I, C and T) present in skeletal and cardiac muscle. Troponin C is identical in both muscle types, but the genes encoding troponin I and T in cardiac and skeletal muscles are different. Increased plasma concentrations of cardiac troponins indicate lysis of cardiomyocytes. Thus, troponins I and T were first studied as very sensitive markers of acute coronary syndromes [19].

During ST-elevation myocardial infarction (STEMI), cardiac troponins are also of prognostic value, as their concentration is directly linked to ischemic myocardial mass. This is also the case in non-STEMI, where the troponin concentration predicts an unfavorable outcome, including CS [20] and often triggers an early invasive management [21].

During CS, plasma troponin concentrations are mainly used for diagnostic purposes, particularly in detecting myocardial infarction as the underlying cause [22, 23]. However, their diagnostic value is often limited by a lack of specificity, as increased plasma troponin I and T concentrations can also result from non-cardiac conditions in critically ill patients, such as sepsis and septic shock [24], respiratory failure [25], or acute kidney injury [26]. Troponin concentrations also have prognostic value and may be useful in predicting failure of veno-arterial extracorporeal membrane oxygenation (VA-ECMO) weaning in refractory CS following myocardial infarction [27].

### B-type natriuretic and n-terminal pro-B-type natriuretic peptides

B-type natriuretic peptide (BNP) is secreted by cardiomyocytes following an increase in the cardiac chamber wall stress [28]. BNP is synthesized as a pro-hormone (pro-BNP), which is cleaved in a 32-amino-acid carboxy-terminal portion (active BNP) and a 76-amino acid amino-terminal portion (N-terminal pro B-type natriuretic peptide, NT-Pro-BNP). It is the carboxy-terminal part that causes natriuresis, diuresis, vasodilation and relaxation of smooth muscles [28].

In cardiology, BNP and NT-pro-BNP were first used as diagnostic markers, in particular to detect a cardiac cause of acute dyspnea [28]. High plasma concentrations have also been described in systolic and diastolic left ventricular dysfunction, in right heart failure as well as during pulmonary embolism or primary pulmonary hypertension [29]. Their prognostic role was then highlighted, for example in congestive heart failure [30]. NT-pro-BNP is also an important prognostic index after STEMI [31], particularly in diabetic patients [32].

In patients with shock, the value of BNP and NT-pro-BNP in diagnosing a cardiogenic cause is disappointing. Although some studies have shown that a low value of NT-pro-BNP had a high negative predictive value [33], others have shown less clear results [34] as in patients with respiratory distress [35]. This is probably due to the numerous extracardiac causes of BNP or NT-pro-BNP elevation, such as sepsis and septic cardiomyopathy, acute kidney injury, obesity and even age and biological sex [30, 36, 37]. On the other hand, the values of BNP and NT-pro-BNP have a prognostic value during CS [34] as during other types of shock [38]. Among patients with acute myocardial infarction, those with NT-pro-BNP concentrations above the median plasma concentration had a significantly impaired clinical course, even if coronary revascularization was successful [39].

### Soluble suppression of tumorigenicity 2 receptor

The soluble suppression of tumorigenicity 2 receptor is a member of the interleukin 2 toll-like receptor superfamily [40]. Two distinct forms are produced, one soluble (sST2) and the other transmembrane. The soluble form, which can be measured in the blood, is a marker of inflammation and fibrosis in cardiac tissues [41, 42]. sST2 binds in vivo to interleukin-33, which is known for its anti-hypertrophic and anti-fibrotic effects on cardiomyocytes. During chronic heart failure, sST2 has a prognostic value [43], but its superiority to well-established prognostic markers is not well established [44].

Plasma sST2 concentrations are higher in patients with cardiogenic shock compared to those with heart failure without shock [30]. This could be linked to stimulation by IL-1β, IL-6 or TNF-α, reflecting the intense inflammatory activity during CS (see below). However, the value of sST2 for diagnosing CS is low as its plasma concentration also increases in other pathological situations, such as cancer or sepsis [45]. Its interest may be more prognostic than diagnostic. Compared to the CardShock clinical score, the combination of NT-pro-BNP with sST2 improved the prediction of mortality in patients with CS due to acute coronary syndrome, whereas neither did so individually [46].

## Dipeptidyl peptidase-3

Dipeptidyl peptidase-3 (DPP3) is a ubiquitous intracellular enzyme that is generally confined within cells but can be released into the bloodstream upon significant cell injury or death [47]. Once released, circulating DPP3 (cDPP3) degrades enkephalins and angiotensin-2 [47, 48]. High plasma concentrations of cDPP3 increase the cleavage of vasoactive peptides and can lead to circulatory failure in particular through a myocardial depressant effect [49].

DPP3 is one of the most interesting cardiac biomarkers described in recent years. In a pivotal study, the authors showed that higher plasma concentrations of cDPP3 are associated with mortality in patients with CS (Table 1) and that intravenous administration of DPP3 results in rapid and profound negative inotropic action in healthy mice [49]. In the same study, the authors reported that in a mouse model of heart failure, an inhibitory antibody against cDPP3, procizumab, reversed cardiac and renal dysfunction [49].

**Table 1:** Summary of pivotal studies and trials for new biomarkers in cardiogenic shock

Study	Date	Patients	Interventional group	Control group	Primary endpoint	Main results
**Dipeptidyl peptidase-3**						
Post-hoc analysis of CardShock study [49]	2020	174 patients with cardiogenic shock and cDDP3 measurements on ICU admission and during ICU stay	–	–	Association between cDPP3 levels and 90 day mortality	cDPP3 levels on admission are predictive for 90 day mortality Decrease in cDPP3 concentration was associated with lower mortality, a decreased need for cardiovascular support and an improvement of renal function
Post-hoc analysis of SPUM-ACS study [51]	2023	4787 patients with ACS and cDDP3 measurements on ICU admission and within the first 24 h after ICU admission	–	–	Association between cDPP3 levels and the occurrence of cardiogenic shock	cDPP3 levels are associated with in-hospital cardiogenic shock occurrence cDDP3 levels are associated with 30 day and one-year mortality Persistent elevated cDPP3 levels are associated with 13.4-fold increased 30 day mortality risk
Ancillary study of Optima-CC trial [52]	2020	57 patients with cardiogenic shock related to AMI and cDDP3 measurements on ICU admission, 24 h, 48 h and 72 h	Epinephrine	Norepinephrine	Association between cDPP3 levels and the occurrence of refractory cardiogenic shock	Patients with higher cDPP3 levels on admission More frequent refractory cardiogenic shock Lower cardiac index Lower estimated glomerular filtration rate Better outcomes in case of rapid decrease in cDPP3 levels
**Biomarkers linked to endothelial dysfunction**						
*Adrenomedullin*						
Post-hoc analysis of CardShock study [58]	2017	178 patients with cardiogenic shock and adrenomedullin measurements on ICU admission and during ICU stay	–	–	Association between adrenomedullin levels and 90 day mortality	Adrenomedullin levels on admission are predictive for 90 day mortality High levels of adrenomedullin are associated with impaired cardiac index, mean arterial pressure, central venous pressure and systolic pulmonary artery pressure Persistent elevated adrenomedullin levels at 48 to 96 h are associated with persistent impaired cardiac and end‐organ function
ACCOST-HH trial [61]	2022	150 patients with cardiogenic shock	Adrecizumab	Placebo	Number of days up to day 30 without the need for cardiovascular organ support	No difference in the number of days without the need for cardiovascular organ support No difference in 30 day mortality No difference in 90 day mortality No difference in adverse effects
*Angiopoietin-2*						
Sub-study of the IABP-SHOCK II trial [65]	2015	189 patients with cardiogenic shock related to AMI and angiopoietin-2 measurements at Day-1, Day-2 and Day-3	IABP	No IABP	30 day mortality	Angiopoietin-2 blood levels are independent predictors for 30 day mortality and 1 year mortality

ACS: acute coronary syndrome; AMI: acute myocardial infarction; cDPP3: circulating Dipeptidyl peptidase-3; IABP: intra-aortic balloon pump; ICU: intensive care unit

An ancillary analysis of the FROG-ICU study found that patients in shock (not just cardiogenic) had higher plasma cDPP3 concentrations than patients without shock [50]. Higher concentrations of cDPP3 were observed in patients who developed acute renal failure or required renal replacement therapy. Finally, there was an association between cDPP3 concentrations on admission and discharge from the intensive care unit and mortality rates, regardless of the type of shock [50].

Similar results were observed in a more specific CS population [51, 52] (Table 1). High baseline plasma cDPP3 concentrations are associated with a more severe prognosis, independent of other prognostic factors, and a greater risk of refractory shock [52]. Increase in cDPP3 concentration is also of great prognostic value. In a large study, compared to normal values, plasma cDPP3 concentrations constantly increased between 12 and 24 h were associated with a 13.4-fold increase in 30 day mortality risk [51].

Therefore, DPP3 is a biomarker indicative of the severity of the consequences of CS. More importantly, it could act as a potential pharmacological target to improve the outcomes of CS through its selective blockade.

## Biomarkers linked to endothelial dysfunction

### Adrenomedullin

**Fig. 1:**
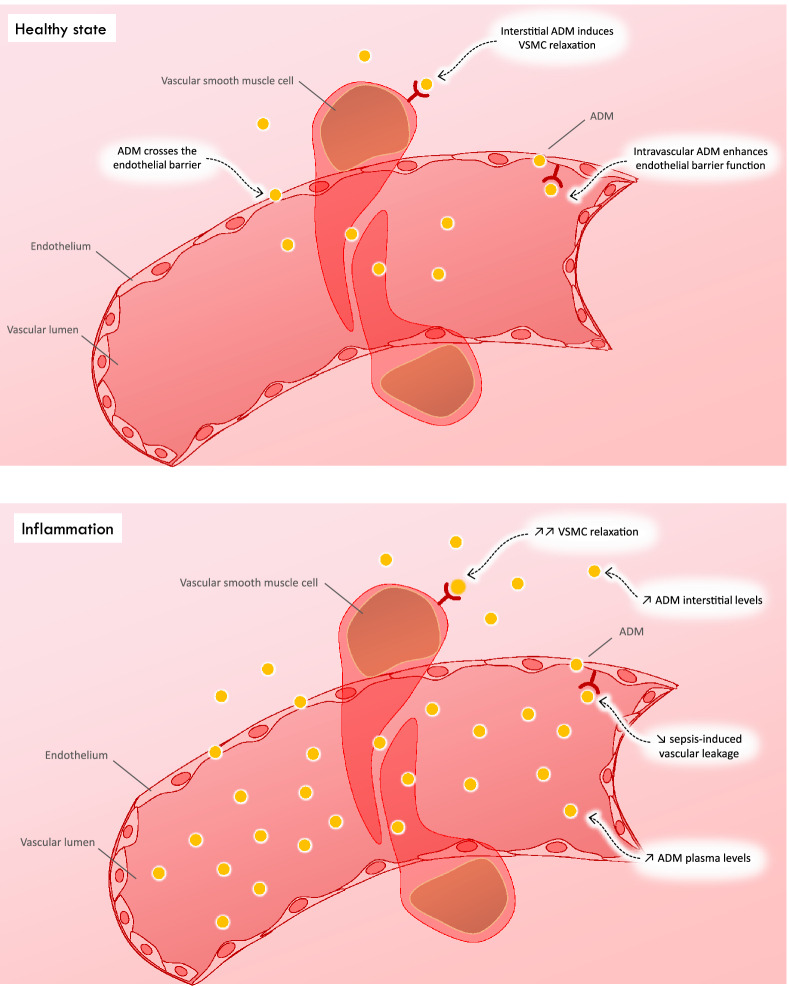
Vascular effects of adrenomedullin in physiological and inflammatory conditions. In physiological conditions, adrenomedullin (ADM) is present in blood and freely crosses the endothelial membrane. In the vascular space, it regulates barrier function and permeability of endothelial cells. In the interstitium, it relaxes the vascular smooth muscle cells (VSMC), inducing vasodilation. In inflammatory conditions, the ADM concentration is increased in plasma and interstitium. In plasma, ADM counteracts vascular leakage, which may be a protective mechanism. In the interstitial space, excess ADM leads to exaggerated vasodilatation

Adrenomedullin (ADM) is a small peptide hormone secreted by various tissues, including vascular smooth muscle cells and endothelium, particularly in pathological conditions such as sepsis [53]. ADM exhibits distinct functions depending on its location (intravascular or extravascular) and target cells (endothelial or vascular smooth muscle cells) [53] (Fig. 1).

Intravascularly, ADM improves the protective anti-permeability properties of blood vessels by regulating the endothelial actin-myosin cytoskeleton. Conversely, ADM interacts with vascular smooth muscle cell receptors located outside the vessels, inducing vasodilation. Although beneficial under physiological conditions, its vasodilatory action can exacerbate arterial hypotension and shock (Fig. 1). ADM may also exert an inotropic effect mediated by cyclic adenosine monophosphate [54].

Because of its role in vasodilation and endothelial permeability, clinical applications of ADM have mainly been explored during septic shock. Its concentration is correlated with the severity of septic shock and patients’ vasopressor needs [55, 56], including if cardiac dysfunction is associated [57]. In a subpopulation of the CardShock cohort, including patients with CS, this prognostic role of ADM was also demonstrated [58]. High plasma ADM concentrations above the population median were associated with twice higher 90 day mortality than lower concentrations [58] (Table 1). This elevation was not related to its inotropic effects but to impaired endothelial permeability. Indeed, left ventricular ejection fraction was identical whether ADM concentration was high or low in patients after acute myocardial infarction [59].

Adrecizumab, a non-neutralizing antibody that binds to ADM, increases the size of the molecule, preventing its migration into the interstitium and increasing circulating concentrations [53]. Adrecizumab stabilizes barrier function and decreases the vasodilatory effects of ADM on vascular smooth muscle cells [53]. During septic shock, in the AdrenOSS-2 trial, adrecizumab caused a larger decrease in the sequential organ failure assessment (SOFA) score than placebo in patients with high plasma ADM concentration, without affecting mortality [60].

In CS, adrecizumab was compared to a placebo in the multicenter and randomized ACCOST-HH trial including 150 patients hospitalized for CS in the last 48 h. Of note, there was no selection based on plasma ADM concentration [61]. The number of days until day 30 without the need for cardiovascular organ support did not differ between the groups [61] (Table 1). Despite this negative result, this biomarker is, with DPP3, one of the most interesting in CS and perhaps even more probably in septic shock: not only does it have a significant prognostic value, but also it can be specifically blocked by an antibody which can be administered in humans.

### Angiopoietins

**Fig. 2:**
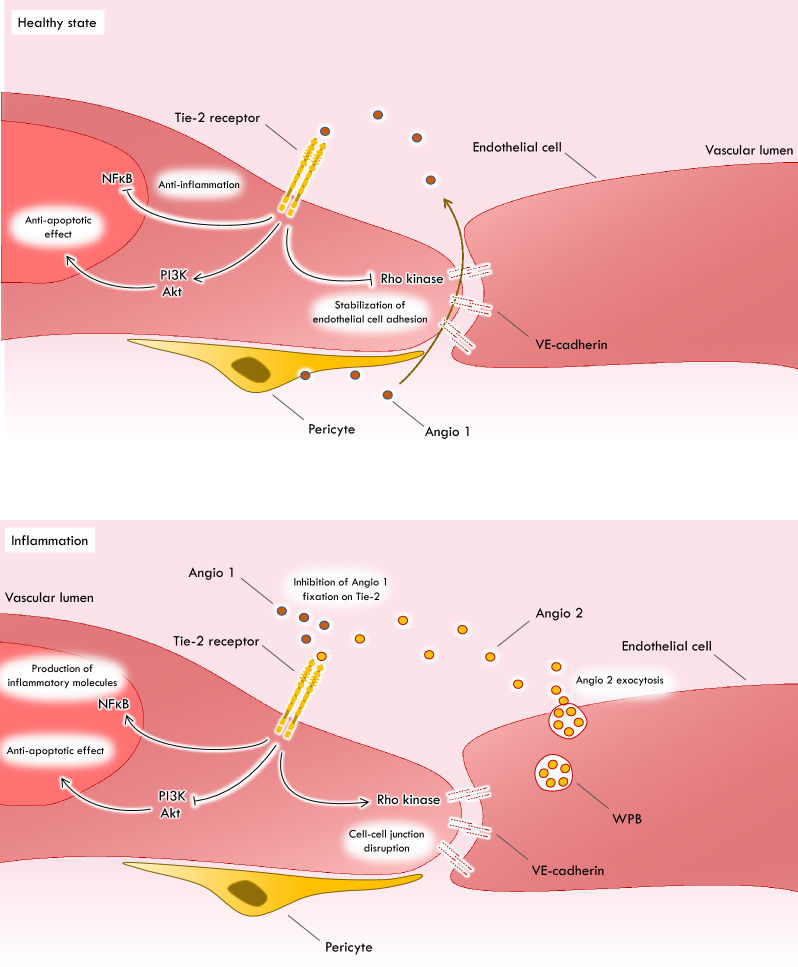
Vascular effects of angiopoietin in physiological and inflammatory conditions. In physiological conditions, angiopoietin-1 (Angio 1) is produced by the pericytes. Angio 1 binds the tyrosine kinase (Tie-2) receptor in the vascular lumen, enhancing intracellular signaling pathways. It activates the PI3K/Akt cell pathway, leading to vascular stabilization. It inhibits transcription of some genes, like angiopoietin-2 (Angio 2). Inhibition of nuclear factor (NF) kB cells suppresses expression of inflammatory genes. It also inhibits the Rho-kinase pathway, which induces disruption of the cell–cell junctions. In inflammatory conditions, various inflammatory agents induce the release of Angio 2 from Weibel-Palade bodies (WPBs). Angio 2 acts as an antagonist of Angio 1, stops Tie2 signaling, and sensitizes the endothelium to inflammatory mediators. This induces the disruption of cell–cell junctions via the Rho kinase pathway, which facilitates capillary leakage

Angiopoietins and tyrosine kinase (Tie)-2 receptor form an endothelial signaling pathway involved in endothelial inflammation, vascular hemostasis, vascular tone, and endothelial barrier function [62]. Activation of the Tie-2 receptor promotes endothelial barrier stability via its effects on cell–cell junctions and the actin cytoskeleton (Fig. 2). Angiopoietin-1 is the main agonist of the Tie-2 receptor. Angiopoietin-2 functions as both an agonist and antagonist of the Tie-2 receptor. Under physiological conditions, it participates in vascular homeostasis (Fig. 2). During inflammation, the plasma concentration of angiopoietin-2 secreted by endothelial cells increases, and angiopoietin-2 becomes an antagonist of the Tie-2 receptor and promotes endothelial permeability and vascular permeability [62] (Fig. 2). Therefore, plasma angiopoietin-2 concentrations, as well as the angiopoietin-2/angiopoietin-1 ratio, may serve as a measure of endothelial barrier function.

As for ADM, it is mainly in septic shock that the role of angiopoietin-2 has been studied. In this context, there is a direct association between plasma angiopoietin-2 concentrations, mortality, pro-inflammatory cytokines [63], severe acute kidney injury and development of acute respiratory distress syndrome [64].

During CS, in a subpopulation of the IABP-SHOCK II trial, plasma angiopoietin-2 concentrations were associated with mortality at 30 and 90 days, including in multivariate analysis [65] (Table 1). During septic shock, experimental studies have tested treatments with neutralizing antibodies to recombinant angiopoietin-1, or angiopoietin-2 targeting short interfering ribonucleotide acids, to improve endothelial barrier function and survival [66]. These strategies have not been investigated in CS at present.

### Endothelin-1

Endothelins are a family of 21 amino acid peptides with three distinct isoforms: endothelin-1, 2, and 3. Endothelin-1 is the predominant isoform that is primarily produced by vascular endothelial cells and induces powerful vasoconstriction by activating two receptors (ET-A and ET-B) [67]. The ET-A receptor is primarily located on vascular smooth muscle cells, and mediates vasoconstriction, while the ET-B receptor is primarily located on endothelial cells and causes vasodilation [68]. ET-1 is involved in several aspects of the pathogenesis of acute heart failure, including decreased cardiac output, vasoconstriction, and neurohormonal activation. It promotes sodium and water retention and participates in myocardial ischemia [69].

During acute heart failure, ET-1 is independently correlated with 180 day mortality, providing additional prognostic information compared to that obtained by NT-pro-BNP [70]. Its plasma concentration decreases with the therapeutic stabilization of patients within 30 days [71]. The precursor of ET-1, pre-pro-endothelin, has the same prognostic value [72]. On the other hand, to date, no study has investigated the place of ET-1 in the specific context of CS, in particular its prognostic value.

## Markers of inflammation

The association between inflammation and cardiovascular disease is well established. The immune response to myocardial infarction has, for example, been well described [73]. During CS, there is added significant activation of inflammation following the release of substances activated by prolonged ischemia of tissues and their reperfusion. The occurrence of a concomitant infection, for example during a digestive translocation, is sometimes involved [74]. Intended to be restorative, the “host response” during CS is excessive or dysregulated and becomes detrimental [74]. The consequences for the different organs, starting with the microcirculation and induced vasodilation, mean that CS takes on the macro and microcirculatory features of septic shock.

An increasing number of biomarkers of systemic inflammation have been reported to describe this host response during CS. The data come largely from sub-studies of randomized controlled trials, examining the prognostic value of various biomarkers in the immune and inflammatory response and mechanistic data are much more limited. In addition to sST2 (see above), classic markers of inflammation, such as leukocyte count and C-reactive protein (CRP), are well-known prognostic factors for chronic heart failure and myocardial infarction [75]. Although this has been less studied, it is undoubtedly also the case in patients with CS. High CRP concentrations have poor prognostic value and are associated with more severe hypoperfusion in patients with CS [76]. An increase in CRP concentrations of at least 200% from day 1 to day 3 during stay in the intensive care unit was associated with an increased risk of 30 day all-cause mortality [77]. The simple leukocyte count also has prognostic value in CS complicating myocardial infarction [78].

In recent years, many new inflammatory biomarkers have also been described as new candidates from molecular signatures in CS, thanks to advances in genomic, transcriptomic, and proteomic data [79, 80]. Soluble urokinase-type plasminogen activator receptor (suPAR), a biomarker reflecting activation of the systemic immune system [81], osteoprotegerin (OPG), a protein of the tumor necrosis factor superfamily, growth differentiation factor 15 (GDF-15), a transforming growth factor β-cytokine, or glucagon-like peptide-1, a gut-derived peptide secreted in response to nutritional and inflammatory stimuli [82] and galectin-3 (Gal-3), a chimeric member of the lectin family involved in numerous biological processes, such as the control of cell–cell and cell–matrix interactions, proliferation, apoptosis, immunity and inflammation [83] have been described to be associated with mortality or to improve the risk stratification of patients with CS. None of these other new biomarkers are yet used in clinical practice.

Finally, some inflammatory biomarkers can be combined to form proteomic scores. The CS4P score, a new protein-based score, combines 4 proteins (liver fatty acid-binding protein, beta-2-microglobulin, fructose-bisphosphate aldolase B and serpinG1) which are not present in the heart, but which are markers of inflammation and the immune response, and which have been found to be associated with the short-term prognosis in CS [84].

In the future, rather than focusing on inflammatory biomarkers, genetic mutations could be studied directly to predict the prognosis of patients with CS. In this regard, it has been reported that patients with CS have a higher frequency of genetic mutations in stem cells that cause clonal hematopoiesis than ambulatory patients with heart failure who are matched on many criteria. This anomaly was associated with poorer survival and higher concentrations of circulating inflammatory cytokines [85].

### Using biomarkers to define subphenotypes

CS is a very heterogeneous syndrome. This heterogeneity can partly explain the neutral results of studies investigating therapeutic measures when applied to a general CS population. Even VA-ECMO did not improve survival in a recent meta-analysis [86]. However, therapeutic strategies may demonstrate a clear benefit if they are applied to subgroups of patients with homogeneous characteristics [87].

Biomarkers can participate in the definition of these subgroups by introducing criteria linked to pathobiological mechanisms. This is called subphenotyping. Biomarkers can, within subgroups of patients, reveal “treatable traits”, *i.e.*, specific biological signatures characteristic of endothelial dysfunction, activation of inflammatory cells, immune or neurohormonal dysregulation [87]. Several studies have attempted to describe clusters of patients, by combining several characteristics, often using artificial intelligence. They were validated by comparing mortality between the different clusters in cohorts of patients with CS.

For example, with a k-means clustering technique, in a multicenter population of patients with CS, a study described 3 phenotypes determined by 6 laboratory variables at admission (white blood cell and platelet counts, glomerular filtration rate, ALT, lactate, bicarbonate), called “non-congestive”, “cardio-renal” and “cardio-metabolic” [88]. The risk of in-hospital mortality and of developing serious shock was greatest in the “cardio-metabolic” group. These results were replicated [89, 90]. The clusters differed in terms of not only one-year mortality, but also echocardiographic characteristics [89].

In another study including a cohort of 21,925 patients with CS [91], an unsupervised machine learning consensus clustering analysis revealed two clusters, different in terms of blood pressure, renal function, different laboratory variables and severity scores. Mortality and incidence of acute kidney injury differed between the two groups [91].

Similarly, two clusters could be identified in the subpopulation of patients with CS from the FROG-ICU study who were discharged alive from hospital [92]. Patients with phenotype B were more anemic and had higher plasma lactate concentrations, persistent renal insufficiency, and persistent elevation of plasma markers of inflammation, myocardial fibrosis, and endothelial dysfunction compared with phenotype A. After adjustment for traditional risk factors, phenotype B was independently associated with 1 year mortality [92]. However, these phenotypes remain very heterogeneous. Other clinical variables could be added, such as those reflecting vascular reactivity and tissue perfusion [93].

### Using biomarkers in clinical practice

For clinical practice, biomarkers should ideally help in the diagnosis of CS, guide the choice between therapeutic options, monitor their efficacy and follow the disease course better than clinical or echocardiographic criteria. “Old” biomarkers satisfy many of these needs. Markers of tissue hypoperfusion (lactate, markers of organ function) help diagnose circulatory failure. Together with arterial hypotension, they classify the patient in stage “C” of the Society for Cardiovascular Angiography & Interventions (SCAI) [94]. They prompt infusion of inotropic agents and monitor the patient's hemodynamic status. They detect deterioration that classifies patients in the SCAI stage “D”. They are used to guide therapeutic options, as for instance weaning from VA-ECMO [95].

The new biomarkers have all been validated by demonstrating their ability to predict the prognosis of patients with CS, but this gives them limited clinical interest. As described above, they could in the future help to better define subphenotypes justifying specific treatments, but there is currently no clinical application. Their interest may stem from the definition of subphenotypes, as described above. An ideal patient phenotyping algorithm should impact clinical decision making, use commonly and quickly obtained variables, and provide additional value to current scores for classifying disease severity (SOFA and APACHE). We are far from this during CS. Future studies should investigate whether specific interventions (vasopressors, inotropes, acute mechanical circulatory support) should be adapted to the phenotype of the patient and improve prognosis compared to current care.

## Conclusion

In patients with CS, “old” biomarkers have been used for decades to detect and measure the severity of acute circulatory failure and cardiac damage, and to measure the effects of treatments. In recent years, many new biomarkers have demonstrated their ability to predict outcomes during CS. This attests to their pathophysiological value, but this is not of great interest for patient care. However, some of them such as cDDP3 and ADM, could represent therapeutic targets with blocking antibodies. Future biomarkers will appear in the years to come, such as transcriptomic markers, which have been little studied until now. These future biomarkers could enable the advent of precision medicine, as it is beginning to emerge in oncology, and thus improve the prognosis of CS, which still has a high mortality rate.

## Data Availability

Not applicable.

## Abbreviations

ADM: Adrenomedullin
ALT: Alanine amino-transferase
AST: Aspartate amino-transferase
BNP: B-type natriuretic peptide
CRP: C-reactive protein
CS: Cardiogenic shock
DPP3: Dipeptidyl peptidase-3
NT-pro-BNP: N-terminal pro-B-type natriuretic peptide
SCAI: Society for cardiovascular angiography & interventions
SOFA: Sequential organ failure assessment
sST2: Soluble suppression of tumorigenicity 2 receptor
STEMI: ST-elevation myocardial infarction
Tie-2 receptor: Tyrosine kinase-2 receptor
VA-ECMO: Veno-arterial extracorporeal membrane oxygenation

## Declarations

Ethics approval and consent to participate.

Not applicable.
